# Promyelocytic Leukemia Protein (PML) Regulates Stem Cell Pluripotency Through Novel Sumoylation Targets

**DOI:** 10.3390/ijms26031145

**Published:** 2025-01-28

**Authors:** Syrago Spanou, Takis Makatounakis, Chrysa Filippopoulou, Georgios Dougalis, George Stamatakis, Christoforos Nikolaou, Martina Samiotaki, Georgia Chachami, Joseph Papamatheakis, Androniki Kretsovali

**Affiliations:** 1 Department of Biology, University of Crete, 71500 Heraklion, Greece; 2 Institute of Molecular Biology and Biotechnology (IMBB), Foundation for Research and Technology-Hellas (FORTH), 70013 Heraklion, Greece; 3 Laboratory of Biochemistry, Faculty of Medicine, University of Thessaly, Biopolis, 41500 Larissa, Greece; 4 Institute for Bio-Innovation, Biomedical Sciences Research Center “Alexander Fleming”, 16672 Vari, Greece

**Keywords:** embryonic stem cells (ES cells), promyelocytic leukemia protein (PML), proteomics, small ubiquitin-like modifier (SUMO), spalt homology 1 (SALL1), cell division cycle associated 8 (CDCA8)

## Abstract

The promyelocytic leukemia protein (PML) and its associated nuclear bodies have recently emerged as critical regulators of embryonic stem (ES) cell identity. Despite their recognized importance, the complete spectrum of PML-mediated molecular events in ES cells remains unclear. In this report, we study how PML is shaping the proteomic and SUMO proteomic landscape in ES cells. Proteomic profiling of PML-depleted ES cells uncovered a downregulation of self-renewal factors and an upregulation of proteins associated with translation and proteasomal activity, reflecting a cellular transition from pluripotency to differentiation. Importantly, PML promotes the sumoylation of pluripotency-related factors, chromatin organizers, and cell cycle regulators. We identified SALL1 and CDCA8 as novel PML-directed sumoylation targets, both critical for ES cell maintenance. SALL1 sumoylation increases the activation of the Wnt pathway, contributing to its ability to inhibit ES cell differentiation. Similarly, CDCA8 sumoylation enhances its capacity to promote cell proliferation. Collectively, our findings demonstrate that PML regulates ES cell identity by modulating the abundance or sumoylation of key regulators involved in pluripotency and cell cycle progression.

## 1. Introduction

Embryonic stem cells (ES cells) possess the unique ability of self-renewal and differentiation into various cell types, a characteristic termed pluripotency. The orchestration of pluripotency is governed by intricate networks of transcription factors, signaling pathways, and epigenetic complexes [1]. Notably, PML (promyelocytic leukemia protein) and its associated nuclear bodies (PML-NB) have emerged as essential regulators of these cellular processes. PML has been shown to safeguard the naive pluripotency state by promoting cell cycle progression and inhibiting the onset of primed pluripotency [2], a role distinct from its well-known function as a tumor suppressor. The diversity of cellular processes regulated by PML-NBs is driven by variations in the client proteins associated with the PML across different cell types or in response to specific stimuli. Intriguingly, PML bodies are reportedly involved in the regulation of numerous, often contrasting cellular processes, including transcription, apoptosis, genome stability, cell cycle control, senescence, stress response, oncogenesis, and stem cell pluripotency [3,4].

PML’s ability to regulate transcription is quite complex and has not been thoroughly explored. PML bodies can both activate and repress transcription by modulating the activity of coactivators or corepressors [5]. Another mechanism involves the relocation of specific gene loci close to PML bodies for transcriptional activation [6] and the establishment of gene activation memory [7]. Recently, a new mechanism was reported for PML bodies in the activation of a gene cluster in the Y chromosome by prohibiting the access of Dnmt3a and DNA methylation [8]. An alternative PML-mediated way to regulate gene expression and cell physiology is by interactions and/or posttranslational modifications (such as phosphorylation, acetylation, or sumoylation) of a large group of proteins collectively named PML–NB clients.

Although several studies have reported transcriptomic changes in embryonic stem cells following the ablation of PML [2,8,9], the corresponding changes at the proteomic level remain largely unexplored. To address this issue, in this study, we undertook the analysis of the changes in ES cell proteome that are imposed by PML loss. Considering the critical role of post-translational modifications in protein function, we aimed to complement our proteomic studies with an examination of PML-dependent changes in sumoylation, an important modification that is particularly controlled by PML-NBs [3].

PML functions are accomplished following its sumoylation-dependent assembly into PML NBs, which allows the subsequent recruitment of client proteins via association with their SUMO Interacting Motifs (SIM) [10,11]. Moreover, the sumoylation of chromatin proteins has been reported to play a significant role in the maintenance of cell identity by inhibiting cell state conversions between somatic and pluripotent stem cells [12]. Despite the recognized importance of PML in stem cell biology, the role and number of proteins sumoylated in a PML–dependent way have not been thoroughly explored.

In this study, we report the comprehensive characterization of the PML-dependent proteome and SUMOylome in embryonic stem (ES) cells. By integrating these data with biochemical analyses, we sought to identify novel protein factors and modifications supported by PML within the context of self-renewing pluripotent stem cells. Our results demonstrate that PML positively regulates the abundance of proteins required for self-renewal, whereas it exerts a negative effect on proteins that are related to translation and proteasome. PML is necessary for the sumoylation of many critical cellular factors that are required for pluripotency maintenance, chromatin organization, and cell cycle progression. Among them, we singled out SALL1 and CDCA8, which play crucial roles in maintaining the undifferentiated state by promoting the Wnt pathway and facilitating cell cycle progression, respectively. These newly identified PML-dependent sumoylation targets broaden our understanding of the diverse molecular mechanisms by which PML regulates ES cell identity.

## 2. Results

### 2.1. PML Regulates the Expression of Proteins Involved in ES Cell Self-Renewal, Translation, and Proteasome Activities

We previously demonstrated that PML is essential for the maintenance of ESC naïve pluripotency by regulating the transcription of a large number of genes [2]. The changes in the ES cell proteome caused by the absence of the PML are not known. For this, we sought to analyze the proteome differences between PML knockdown (KD) and wild-type (WT) CGR8 cells.

**Figure 1:**
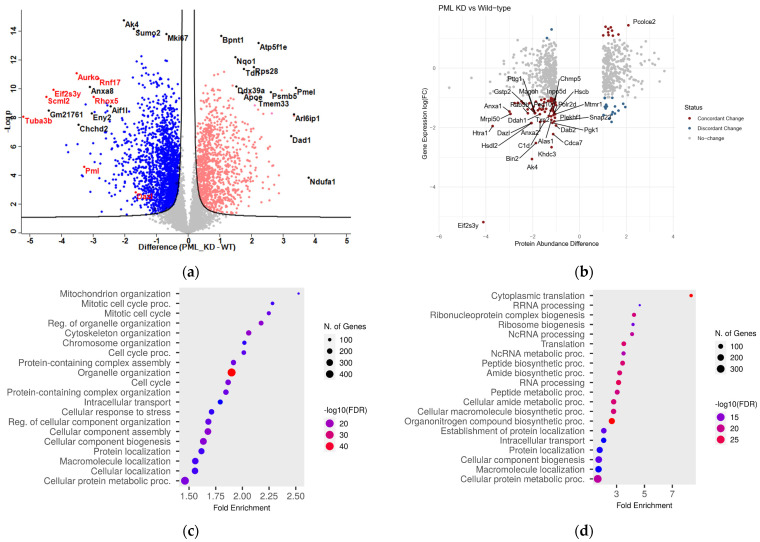
The PML-dependent proteome of ES cells. Comparison with the transcriptome. (**a**) Volcano plot representation of the proteins that are under- or over-abundant (blue and red blots respectively) in the absence of PML (PML KD) (at an absolute cut-off value of 0.5). Highly deregulated proteins are annotated. PML and spermatogenesis-related proteins are colored in red. (**b**) Scatter plot showing the gene expression and protein abundance relative values for 741 genes/peptides with an absolute change > 1 at the protein level. The correlation between gene expression and protein changes was r = 0.23 (Pearson’s correlation coefficient, *p* < = 10-10). Red dots mark genes with concordant changes, blue dots mark genes with discordant changes. The top 30 most divergent genes are annotated. (**c**,**d**) ShinyGO 0.8 representation of GO categories linked to down-(C) or up-(D) regulated proteins in PML KD cells at the <-0.5 and/or >0.5 cutoff.

As shown in Table S1, a total of 6420 proteins were identified. In the absence of PML (PML KD), the number of down-regulated proteins was greater than that of up-regulated ones; 1468 vs. 929 at the threshold of an absolute log2-difference > 0.5 and *p*-values < 0.05. (Table S1). In Figure 1a, proteins that are down-(blue) or up-(red) regulated in the absence of PML (PML KD vs. WT) are depicted. Proteins that were repressed in the absence of PML included Dazl, Scml2, Tuba3b, Eif2s3y, Rnf17, Rhox5, and Aurkc (Figure 1a, marked in red along with PML), which are related to the regulation of spermatogenesis [13,14,15,16]. The link between PML and spermatogenesis needs further investigation. In the category of upregulated proteins, notable examples included proteasome (Psmb5) and mitochondrial (Atp5f1e, Ndufa1) proteins.

To gain a more comprehensive understanding of the biological processes regulated by PML, we compared the PML-dependent proteome with our previously published transcriptomic data [2]. We observed an overall bias for down-regulation at both the RNA and protein levels when comparing PML KD with the WT (Figure S1a), suggestive of a general trend of congruence between transcription and translation levels.

The overall positive correlation of RNA and peptide abundance changes was evident among a core of 96 genes, selected with strict criteria for both differential RNA and protein levels (|log2 difference| > 1 and *p* < 0.05) (Figure 1b). Out of those, we identified 77 highly significant genes with concordant changes (same direction of expression/abundance change in RNA/protein level), compared to 19 with discordant change (different direction of expression/abundance change) (Table S2). Concordantly, suppressed genes (i.e., decreased at both RNA and protein levels) in the absence of PML were functionally enriched for association with RNA PolII transcription (Figure S1b), highlighting the role of PML in gene transcription.

**Figure 2:**
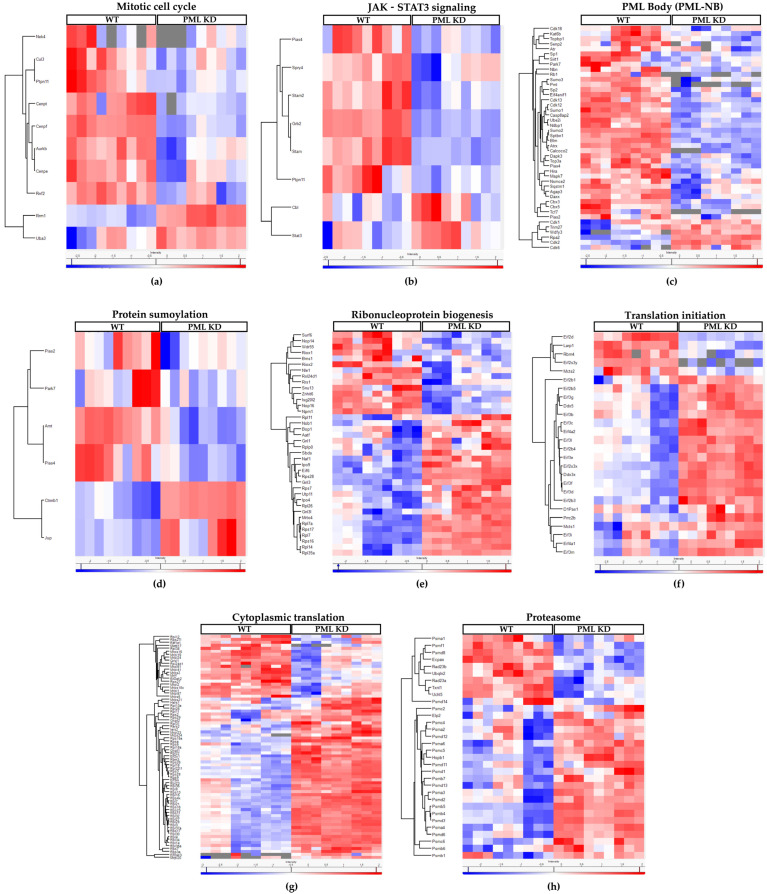
PML regulates the expression of proteins involved in ES cell self-renewal, translation, and proteasome. Hierarchical analysis (Heatmaps) of z-scores for protein abundances in biological processes (GOBP) that are either down- or upregulated in PML KD cells in comparison with the WT. (**a**) Mitotic cell cycle, (**b**) JAK−STAT3 signaling, (**c**) PML body, (**d**) Protein sumoylation, (**e**) Ribonucleoprotein biogenesis, (**f**) Translation initiation, (**g**) Cytoplasmic translation, (**h**) Proteasome. Color key indicates the protein expression value (blue: lowest; red: highest). Each column represents a biological replicate (*n* = 3) for WT or PML KD ESC. Proteins were clustered using the Perseus software (version 1.6.15.0).

A functional analysis of the PML-dependent proteome was performed, focusing on biological processes and employing ShinyGO 0.80 http://bioinformatics.sdstate.edu/go/ (accessed on 21 October 2024). As shown in Figure 1c, the mitotic cell cycle process, along with organelle and chromatin organization, are the top-down-regulated pathways in PML KD cells, in agreement with the growth-promoting role of PML in this cell context [2]. Top-up regulated molecular pathways include translation, ribonucleoprotein, and ribosome biogenesis (Figure 1d). A detailed hierarchical analysis comparing the relative levels of proteins related to the mitotic cell cycle and the JAK/STAT pathway shows strong suppression in PML KD cells (Figure 2a,b), in line with the described role of PML as a cell cycle progression factor and a STAT3 activator in ES cells [2]. Remarkably, a large group of known PML body residents are positively regulated by PML (Figure 2c). Moreover, further functional analysis of the ES proteome revealed a group of sumoylation-process-related proteins (Figure 2d) to be suppressed in PML KD cells in agreement with the importance of PML nuclear bodies for sumoylation [6]. In the group of up-regulated proteins, we observed an enrichment of functions related to ribonucleoprotein complex biogenesis, translation initiation, cytoplasmic translation, and proteasome (Figure 2e, Figure 2f, Figure 2g, and Figure 2h, respectively). These data correlate with the global translational rate and ribosomal biogenesis increase observed when cells initiate differentiation [17]. The upregulation of proteasome subunit proteins may indicate a need to balance the increased translation rates, thereby supporting cellular homeostasis.

Collectively, PML loss causes a decrease in the expression levels of proteins associated with embryonic stem cell self-renewal, including the mitotic cell cycle and JAK-STAT3 processes, PML nuclear body (PML-NB) assembly, and sumoylation. Conversely, it leads to an increase in the expression levels of proteins involved in cellular translation, ribosome biogenesis, and proteasome function. 

### 2.2. PML Promotes the Sumoylation of Key Regulators Involved in ES Cell Pluripotency

**Figure 3:**
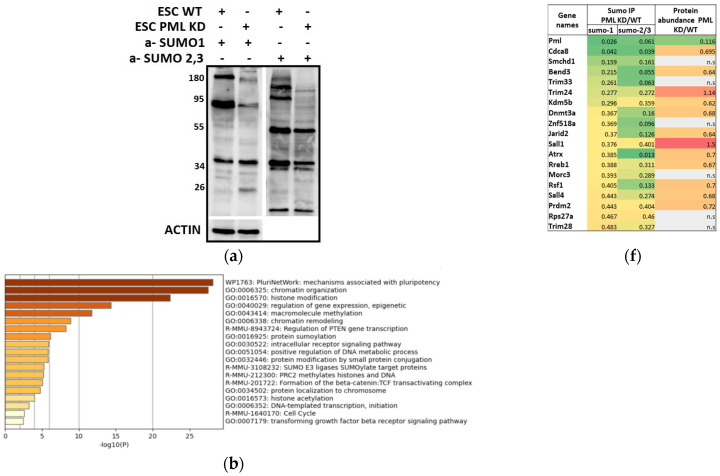

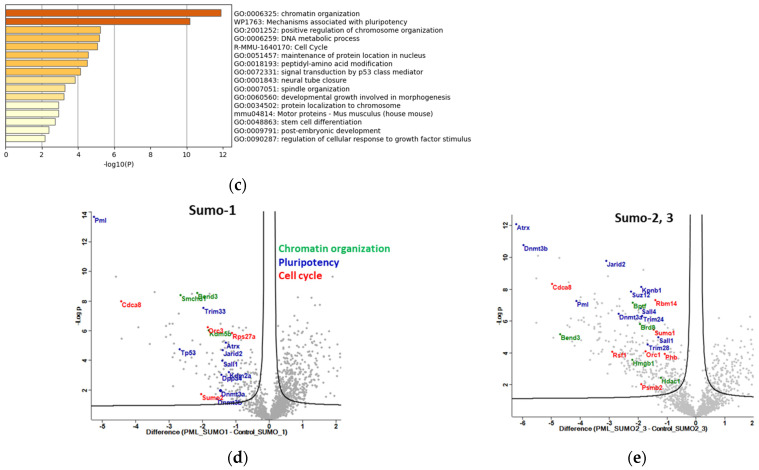
PML promotes the sumoylation of key regulators involved in ES cell pluripotency. (**a**) immuno-detection of bulk SUMO-1 and SUMO-2,3 modified proteins in WT and PML KD cells. WB using anti-SUMO-1 and SUMO-2, 3 antibodies. (**b**,**c**) Enriched terms across genes coding for proteins under-sumoylated in PML KD cells. B. SUMO-1, C. SUMO-2,3 (Metascape). (**d**,**e**) Volcano plots displaying the log_2_ fold change (*x* axis) against the *t* test-derived −log_10_ statistical *p* value (*y* axis) for proteins that are strongly under-sumoylated in PML KD cells, regarding chromatin organization (green), pluripotency (blue), and cell cycle (red), using Perseus software (*n* = 3, FDR = 0.05). Down-regulated proteins are displayed on the left (negative log_2_ fold change values) and up-regulated proteins on the right (positive log_2_ fold change values). (**d**) SUMO-1 (**e**) SUMO-2,3. Green, blue, red denote chromatin organization, pluripotency and cell cycle, respectively. (**f**) Heatmap presenting common SUMO-1 and SUMO-2,3 target proteins that are under-sumoylated in PML KD showing the abundances of SUMO IP in PML KD against WT and the corresponding protein abundances.

To confirm our earlier finding that silencing PML results in the downregulation of sumoylation-process-related proteins (Figure 2d), we investigated whether this silencing also influences global sumoylation levels. Using protein extracts from PML KD ES cells, we detected reduced amounts of SUMO-modified proteins by either SUMO1 or SUMO2/3 compared to the WT cells (Figure 3a), showing the importance of PML for this modification.

Due to our previous observation and because sumoylation is closely associated with the assembly and functions of PML nuclear bodies [6], we chose to identify changes of the SUMOylome between the control and PML knockdown (KD) cells. Sumoylated proteins from the two cell lines were immunoprecipitated using beads coupled with SUMO-1, SUMO-2/3, or IgG (control). Eluted proteins were subjected to proteomic analysis using LC-MS/MS. A complete list of SUMO-1 or SUMO-2,3 target proteins in both cell types is shown in Table S3. Applying a twofold cutoff for PML KD against the WT difference, we detected 155 SUMO-1 and 166 SUMO 2, 3 target proteins (Table S4). In a previous report, the authors employed a His/SUMO-2 overexpression strategy and identified 77 PML-dependent SUMO-2,3 target proteins in E14tg2a ES cells [9]. We compared our data with their list and detected 18 common proteins with our SUMO-2,3 and 7 common with our SUMO-1 list (as shown in Table S5). We also compared our results with another study [18] performed in PML-deficient mouse embryonic fibroblasts, where the authors identified IFNa-induced and PML-dependent sumoylation proteins and detected 47 common proteins (Table S5).

We next performed a functional enrichment analysis of the PML-dependent SUMOylome using Metascape [19]. Down-sumoylated proteins for both SUMO-1 and SUMO-2,3 in PML KD cells included genes primarily related to pluripotency, chromatin organization/histone modifications, and the cell cycle (Figure 3b,c). Proteins showing strong sumoylation variability in the absence of PML are indicated in Figure 3d,e. While down-sumoylated proteins were enriched in the processes mentioned above, those found to be over-sumoylated in PML KD cells were related to mRNA processing mechanisms and diverse functional groups at lower significance levels (Figure S2a).

In order to test whether sumoylated proteins are associated with PML-NB, we compared the groups of SUMO-1 and SUMO-2,3 target proteins (Table S4) with a recently reported list of PML-interacting proteins in mouse ES cells using Turbo-ID [20] (dataset EV2). We observed that the majority of proteins that are strongly sumoylated in PML-expressing cells are PML-NB clients (Figure S2b, 57% of SUMO-1 and 67% of SUMO-2,3 targets), indicating that these proteins are sumoylated upon their association with PML-NB.

In Figure 3f, we display proteins that were under-sumoylated by both SUMO-1 and SUMO-2,3 in the absence of PML, indicating the sumoylation and protein abundance differences of the PML KD against the WT. The majority of them were proteins related to pluripotency and chromatin organization. We next validated the changes in SUMOylation status in WT CGR8 as opposed to the PML KD cells using SUMO-immunoprecipitation combined with immunoblotting (Figure S3a,b). We confirmed the reduction of sumoylation in PML KD compared to the WT cells of selected proteins from Figure 3f, ATRX, SALL1, SALL4, TRIM24, and CDCA8 (Borealin). The reduction of sumoylation was not due to changes in their corresponding protein expression levels (Figure S3a and S3b for SUMO1 and SUMO-2/3, respectively). Thus, as previously mentioned, global protein abundance differences are not sufficient to explain the full extent of sumoylation divergence between the control and PML KD cells.

In conclusion, the capacity of PML to enhance the self-renewal and pluripotency of embryonic stem (ES) cells may be attributed to the sumoylation of important factors that are involved in these processes. Among the proteins depicted in Figure 3f and Figure S3b, we selected CDCA8, which is the top PML-dependent SUMO target, and SALL1. Both proteins are connected to important features of ES cells, CDCA8 to the cell cycle progression and SALL1 to pluripotency.

### 2.3. Sumoylation Increases the Stability and Wnt Pathway Potentiation Activity of SALL1

The Spalt Homology 1 (SALL1) protein is responsible for causing Townes–Brocks Syndrome (TBS), leading to a combination of anal, renal, limb, and ear anomalies [21] through molecular mechanisms that involve its function as a transcription factor and its role in regulating primary cilia functions [22]. SALL1, a member of the pluripotency network of ES cells [21], has been reported to promote ESC pluripotency [22] and contribute to reprogramming into pluripotency [23].

**Figure 4:**
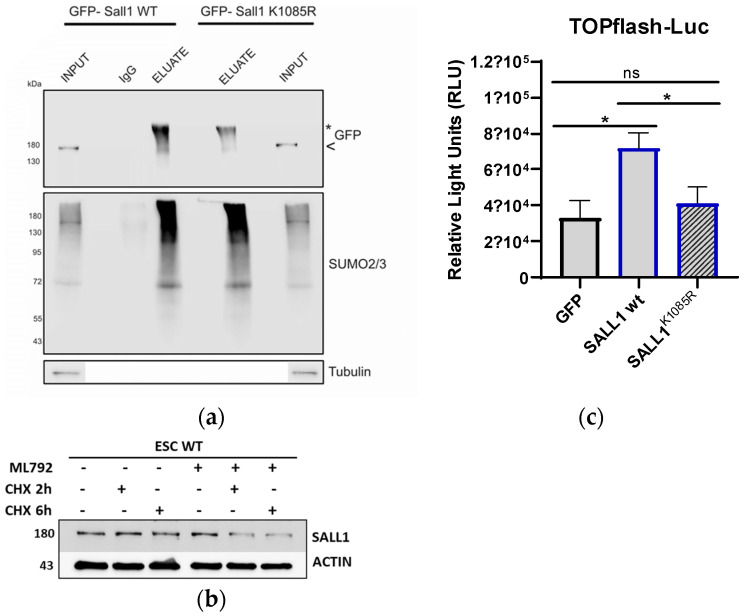
Sumoylation increases the stability and Wnt-pathway-enhancement activity of SALL1. (**a**) The K1085R mutant SALL1 shows decreased sumoylation levels when compared with the WT. SUMO co-IP followed by WB analysis using a−GFP, a−SUMO-1, or a−SUMO-2,3 antibodies. (**b**) The half-life of SALL11 was measured after the addition of 150 μg/mL CHX for the indicated points in the absence or presence of ML−792 (1 μM) for 24 h. Protein extracts from control or treated cells were subjected to WB with the indicated antibodies. (**c**) Luciferase assay using extracts from HEK293 cells transfected with the TOPFlash-luc reporter in the presence of SALL1 WT or K1085R-mutant-expressing constructs. Graph represents mean values ± SEM (*n* = 4, * *p* ≤ 0.0176; two-way ANOVA).

The in vitro sumoylation of human SALL1 by UBE2I at lysine 1086 has been reported [23] and confirmed by later SUMO proteomic analyses [24], but the functional consequences of this modification in ES cells have not been studied. To examine the role of SALL1 sumoylation, we mutated lysine 1085, which was reported to be the major sumoylation site in ES cells [25], to arginine. We confirmed that sumoylation is severely reduced in this mutant (more than 50%) compared to the WT (Figure 4a). We examined whether SALL1 and PML-NBs co-localize in the nucleus. As shown in Figure S4a, there is limited localization of either WT or MUT SALL1 proteins in PML-NBs. We next employed the SAE inhibitor ML-792 in combination with cyclohexamide to test if sumoylation regulates the stability of SALL1. Figure 4b and Figure S4b show that the inhibition of sumoylation reduces the protein’s half-life. The same effect of sumoylation inhibition was noticed in another ES cell line, E14tg2a (Figure S4c,d). SALL11 is a well-characterized activator of the Wnt pathway [26], which is essential for maintaining the pluripotency of embryonic stem (ES) cells. Thus, we then tested the consequence of SALL11 sumoylation on the activation of the Wnt pathway. We used the TOPflash luciferase reporter that measures TCF/LEF transcriptional activity in the Wnt signaling pathway. As shown in Figure 4c, co-transfection with plasmids expressing SALL1 showed that SALL1 WT potentiated luciferase activity to a much higher level relative to the K1085R mutant protein. Thus, sumoylation is a molecular mechanism that regulates both the turnover and the transcriptional activity of SALL1 in ES cells.

### 2.4. Sumoylation Regulates the Stability and the Cell Cycle Progression Activity of CDCA8

The cell division regulator and component of the chromosomal passenger complex, CDCA8/Borealin, is strongly expressed in undifferentiated ES cells and plays an essential role in mouse embryonic development [27,28]. CDCA8 is one of the most important PML-dependent sumoylation targets (Figure 3c,d) and a PML-interacting protein in ES cells [20]. Therefore, we hypothesized that CDCA8 sumoylation [29] could play a role in influencing the cell cycle progression activity of PML in (ES) cells.

We mutated all five lysine residues (63;88;109;124;192) that were reported to be sumoylated in mouse ES cells [25] and fused both WT and sumoylation-mutant (MUT) proteins with GFP. Following the transfection of these proteins along with PMLIV-mCherry in HEK293 cells, we observed that both proteins highly co-localized with PML-NB. Thus, CDCA8 is a PML-NB client protein in ES cells.

**Figure 5:**
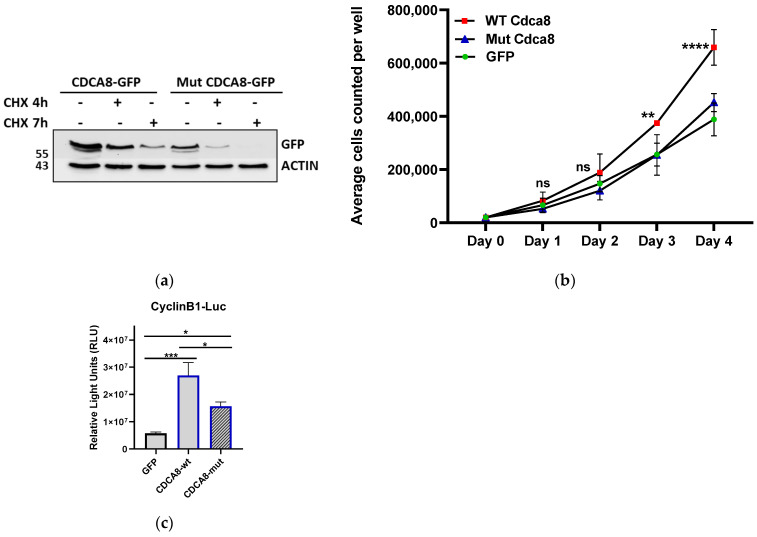
Sumoylation regulates the stability and cell cycle progression activity of CDCA8. (**a**) The half-life of CDCA8, following the addition of CHX for 4 and 7 h on HEK293 stable cell lines expressing GFP/CDCA8 WT and Sumoylation mutant (Mut). Cell extracts were subjected to WB using a-GFP antibody. (**b**) Growth curve of GFP, GFP/CDCA8 WT, and GFP/CDCA8 MUT−expressing cell lines. (*n* = 3, ** *p* ≤ 0.0044, **** *p* < 0.0001, ns = non significant; two-way ANOVA) (**c**) Luciferase assay using extracts from HEK293 cells transfected with the CyclinB1-luc reporter in the presence of CDCA8 WT or sumoylation mutant expressing constructs. Graph represents mean values ± SEM (*n* = 5, * *p* ≤ 0.0487, *** *p* = 0.0007; two−way ANOVA).

WT and sumoylation-mutant (MUT) proteins, as GFP fusions, were next used for the creation of stable cell lines. These cell lines were then treated with cycloheximide in order to determine the respective turnover rates of the WT and the sumoylation-mutant proteins. Immunodetection revealed that sumoylation increases the stability of the CDCA8 protein, as the mutant protein has a shorter half-life compared with the wild type (Figure 5a). Considering the importance of CDCA8 for mitotic progression, we measured the growth curves for the abovementioned cell lines. In analogy with its role in cancer [30], the overexpression of CDCA8 WT caused a higher cell proliferation rate compared to the control (GFP), whereas the sumoylation mutant lost this ability (Figure 5b). CDCA8 was reported to stimulate tumor development of hepatocellular carcinoma (HCC) via the potentiation of cell cycle progression factors, such as cyclins, Ki 67, and PCNA [30,31]. To test whether sumoylation could be involved in this activity, we used a Cyclin B1 promoter luciferase reporter. As shown in Figure 5c, the addition of CDCA8 WT stimulates the activity of the cyclin B promoter, whereas the sumoylation mutant protein cannot. Thus, we conclude that sumoylation is important for the cell cycle progression regulation of CDCA8.

## 3. Discussion

PML nuclear bodies (PML-NB) are dynamic intranuclear hubs that regulate numerous cellular processes, including transcription, apoptosis, stress response, cell cycle progression, antiviral responses, and genome stability [3]. The biochemical mechanisms that mediate the effect of PML on these processes are not easy to dissect, as they involve, in addition to different PML isoforms, divergent sets of client proteins dependent on the cell type and genetic background. Although the role of PML in oncogenesis has been well clarified, the elucidation of PML functions in cell fate decisions is far from complete. The PML-dependent transcriptome in ES cells has been reported by our [2] and other studies [8,9]; however, a proteomic or in-depth post-translational modification analysis is still lacking. Specifically, sumoylation has emerged as a major player in orchestrating networks of proteins that determine cell fate [12]. To bridge this gap, we have conducted an investigation into the proteomic and sumoylation changes in embryonic stem (ES) cells in the absence of PML.

Our proteomic analysis revealed that proteins that are suppressed in the absence of PML were related to mitotic nuclear division, the mitotic cell cycle, and JAK/STAT signaling, pathways that we previously demonstrated to be stimulated by PML in naïve pluripotent stem cells [2]. Comparing our proteomic data with previous transcriptomic data revealed a positive correlation that mainly affects the genes that are downregulated at both the RNA and protein levels. Among these genes, we noticed the occurrence of those related to RNA PolI II transcription. Thus, PML behaves mostly as a gene activator in ES cells. Interestingly, PML-NBs of embryonic stem cells were reported to localize close to gene loci, with features of active transcription such as an open chromatin structure, an enrichment of active histone modification marks, and a depletion of repressive histone modification marks [8]. Moreover, we observed that a group of proteins associated with PML-NB, including ATRX, HIRA, DAXX, and SP1 [6], were expressed in a PML-dependent way, with the majority of them being repressed in the absence of PML. These data point to the hub role of PML-ΝΒ in the regulation of multiple client and non-client genes at the RNA and/or protein levels. Another group of proteins that was strongly repressed in the absence of PML were associated with spermatogenesis (Figure 1a). This finding may indicate a potential role of PML in spermatogenesis [32].

Proteins that were upregulated in PML knockdown (KD) cells were associated with ribosome assembly, translation, and proteasome. An increase in translation rates has been linked to the initiation of embryonic stem (ES) cell differentiation [17]. Our previous research demonstrated that the ablation of PML facilitates the transition of ES cells from a naïve state to an epiblast pluripotency state [2]. Therefore, the upregulation of proteins related to translation and proteasome may represent a compensatory mechanism aimed at sustaining cellular growth and protein synthesis as cells transition out of the undifferentiated state in the absence of PML. Together, these results suggest that PML-mediated regulation of protein synthesis may contribute to the maintenance of the stemness.

The repression of a group of proteins related to sumoylation in PML KD cells led us to further study the PML-dependent SUMOylome. By applying an unbiased SUMO-IP approach, we were able to identify 155 SUMO-1 and 166 SUMO-2,3 proteins that show significant changes in their modification levels in the presence of PML (Table S4). In a similar study employing the His/SUMO-2 over-expression strategy, the authors identified 77 PML-dependent SUMO-2,3 target proteins. They reported that the sumoylation of KAP1 and DPPA2 proteins impedes ES cell transition to a 2C state [9]. Comparing their list with our data, we found 18 in common with our SUMO-2,3 proteins, and also 7 in common with our SUMO-1 list. We consider that our unbiased approach allowed for a higher number of SUMO-2,3 target proteins.

Functional enrichment analysis of the PML-dependent SUMOylome revealed that PML promotes the modification of proteins by SUMO-1 and SUMO-2,3 that are related to pluripotency, chromatin organization, and cell cycle regulation. Therefore, sumoylation is another molecular mechanism employed by PML for the potentiation of stem cell pluripotency [2]. Interestingly, more than 50% of these proteins are PML interactors, indicating that they probably become sumoylated following their association with PML-NB. Our data are in agreement with a previous publication showing that the sumoylation of epigenetic and chromatin regulators maintains the pluripotent stem cell state by inhibiting differentiation [25].

We report here that SALL1 and CDCA8 are two novel PML SUMO targets that promote the undifferentiated state and cell cycle progression, respectively. SALL1 is involved in the regulation of stem cell pluripotency by inhibiting the expression of differentiation-related genes and synergizing with NANOG for transcription activation [33]. We show here that the sumoylation of the SALL1 protein increases its stability and potentiates the ability of SALL1 to activate the Wnt pathway [26], which sustains the ES cell’s undifferentiated state [34]. This function may be partly due to the binding and sequestration of the Wnt negative regulators, TLE and DACH1, which have been reported to interact with sumoylated SALL1 in a recent study using SUMO-ID technology [35]. Therefore, it is likely that SALL1 SUMOylation plays a role in the protein’s capacity to inhibit the differentiation of embryonic stem cells [33].

PML ablation in ES cells triggers a series of molecular events that promote the exit from naïve pluripotency and entry into the primed state. The latter is characterized by a cell cycle extension [2]. CDCA8 is an essential regulator of mitosis and cell division [36], which orchestrates proper chromosome segregation and cytokinesis [37]. CDCA8 has been reported to be a PML-NB client, specifically in ES cells, but not in MEFs [20]. Here, we show that sumoylation not only stabilizes CDCA8 but also potentiates its function to promote cell proliferation. We suggest that the role of PML in regulating cell cycle progression in embryonic stem (ES) cells may be partly attributed to the sumoylation of CDCA8.

## 4. Materials and Methods

### 4.1. Reagents and Antibodies

ML-792 (Selleckchem S8697) was used at 1–2 μM. Cycloheximide (CHX, Sigma-Aldrich, St. Louis, MO, USA) was added at a final concentration of 50–150 μg/mL. The antibodies used were: anti-PML (1:1000, Millipore 05-718, Athens, Greece), anti-SUMO-1 (1:500, sc-5308), SUMO-2, 3 (1:500, sc-393144, Proteintech, Planegg-Martinsried Germany), anti-β actin (1.500, sc-69879), anti-GFP (1:500, sc-9996), Sox2 (1:1000, Cell Signaling #2748, Cell Signaling Technologies, Danvers, MA, USA), Sall1 (1:100, Affinity DF13458, Affinity Biosciences, Cincinnati, OH, USA), Sall4 (1:500, sc-101147) Atrx (1:500, sc-55584), CDCA8/Borealin (1:500, sc-376635), and anti-tubulin (1:1000, DHSB).

### 4.2. Plasmids, Mutagenesis, and Knockdown

For knocking down the PML protein in CGR8 cells [2], we used the PML shRNA-pLKO.1 lentiviral construct provided by Prof. Levi BZ and as described in Khalfin-Rabinovich et al., 2011 [38]. The shRNA includes the sequence 5′-GCACAGATGTGCTCAGCTATA-3′ between nucleotides 787–808 of mouse PML mRNA. The GFP/Sall1 construct has been previously described [33] and used as a template for the construction of the K1085R using-side-directed mutagenesis. Mouse Cdca8 cDNA (aa1 to 289) was produced by RT-PCR using the primers FOR: 5′-ATGGCTCCCAAGAAACGCAGC-3′, and REV: 5′-TCATCGGCCCGTCCGTATGC-3′, and cloned first in pBS (SmaI) and finally in EGFP-C2 (EcoPI-BamH1). Mutant Cdca8 (K63R, K88R, K109R, K124R, K192R) cDNA was from Eurofins Genomics (Mainz, Germany) and cloned in EGFP-C2 using the same restriction sites as the WT. For the production of lentiviruses, WT and MUT Cdca8 cDNAs were cloned in pLenti CMV GFP Puro (658-5), which was a gift from Eric Campeau and Paul Kaufman (Addgene plasmid #17,448) [39]. Lentiviral supernatants were collected following the co-transfection of pLenti plasmids with psPAX2 (Addgene#12260) and pMD2.G (Addgene#12259) gifts from Didier Trono [39]. The TOPflash (TCF Reporter) was from Sigma-Aldrich (21-170) (Sigma-Aldrich, St. Louis, MO, USA). The Cyclin B1-luc reporter was constructed by inserting 484 bp of human cyclin B1 promoter (−367 to +117) in front of the pGL3 basic vector (Promega, Madison, WI, USA). The primers used were: CyclB1-prom-for: TTGCTGCTACCGTAGAAATG and CyclB1-prom-rev: AGCCAAGGACCTACAACCAG.

### 4.3. Cell Culture, Cell Transfections and Generation of Stable Cell Lines

Feeder-independent CGR8 murine ESCs were cultured on 0.2% gelatin in DMEM medium (GIBCO, Waltham, MA, USA) supplemented with 15% Fetal Bovine Serum (FBS) (GIBCO), 0.2 mM β-mercaptoethanol (Applichem, Darmstadt, Germany), 2 mM L-glutamine (GIBCO), 1× MEM nonessential amino acids (GIBCO), and 500 U/mL LIF (ESGRO/Millipore). HEK293T cells were cultured in DMEM medium (GIBCO) supplemented with 10% Fetal Bovine Serum (FBS) (GIBCO), 2 mM GlutaMax (GIBCO), and 50 μg/mL Gentamicin (GIBCO). Cell transfections were performed using the calcium phosphate and Lipofectamine™ 2000 (Thermo Fisher Scientific, Waltham, MA, USA). The PML KD cell line was generated by stably expressing, in CGR8 cells, the PML shRNA-pLKO.1 lentiviral vector. Cells grown to 80% confluence were infected with the lentiviruses produced either by the empty shRNA or the PML shRNA vector for 72 h. The selection of shRNA-infected cells was achieved with puromycin (2 μg/mL). Stable cell lines expressing Lenti/GFP CDCA8 WT and Lenti/GFP CDCA8 Mut were generated through selection with puromycin after 3 days.

### 4.4. Proteomic Data and Analysis

Protein extraction from the two cell lines (PML KD and WT) was performed using a lysis buffer consisting of 4% SDS, 0.1 M DTT, and 0.1 M Tris at pH 7.4. The samples were subjected to heating for 3 min at 99 °C, an incubation for 30 min in a water bath-mediated sonication, followed by a centrifugation step for 15 min at 17,000× *g*. Proteomic sample preparation was performed using the single-pot, solid-phase-enhanced sample preparation Sp3 protocol [40]. Then, 20 μg of beads (1:1 mixture of hydrophilic and hydrophobic SeraMag carboxylate-modified beads, GE Life Sciences) were added to each sample in 50% ethanol. The protein extract was collected and processed using magnetic beads, including an alkylation step in the dark for 15 min in 10 mg/mL iodoacetamide. The proteins were allowed to bind to the beads for 15 min, followed by repeated steps of protein clean-up using a magnetic rack. The beads were washed two times with 80% ethanol and once with 100% acetonitrile (Fisher Chemical, Waltham, MA, USA). The proteins captured on beads were digested overnight at 37 °C under vigorous shaking (1200 rpm, Eppendorf Thermomixer, Thermo Fisher Scientific, Waltham, MA, USA) with 0.5 μg Trypsin/LysC (MS grade, Promega, Madison, WI, USA) prepared in 25 mM ammonium bicarbonate. The following day, the supernatants were collected, and the peptides were purified using a modified Sp3 clean-up protocol and were finally solubilized in the mobile phase A (2% acetonitrile and 0.1% formic acid in water), sonicated, and the peptide concentration was determined through absorbance at a 280 nm measurement using a nanodrop instrument. Three biological replicates and three technical replicates were included in the final dataset.

Orbitrap raw data were analyzed using DIA-NN 1.8.1 software (Data-Independent Acquisition by Neural Networks) [41] by searching against the *Mus musculus* spectral library, containing 21,946 proteins derived from the library-free mode of the software and allowing up to two tryptic missed cleavages. A spectral library was created from the DIA runs and used to reanalyze them. DIA-NN default settings were used along with the oxidation of methionine residues and acetylation of the protein N-termini set as variable modifications and the carbamidomethylation of cysteine residues as fixed modifications. N-terminal methionine excision was also enabled. The match between the runs (MBR) feature was used for all analyses, the output (precursor) was filtered at 0.01 FDR, and finally the protein inference was performed on the level of genes using only proteotypic peptides. The generated results were processed statistically and visualized in the Perseus software (1.6.15.0) [42]. Intensity values were log (2) transformed, a threshold of 70% of valid values in at least one group was applied, and the missing values were replaced from normal distribution (Width: 0.3, Down shift: 1.8). For statistical analysis, Student’s *t*-test was performed, and permutation-based FDR was calculated using a 5% threshold.

### 4.5. Transcriptome vs. Proteome Comparison

Processed and quantified transcriptome data were obtained from [2] (GEO: GSE93922), corresponding to a set of 24,408 protein-coding genes. Proteomics data were derived from this publication and resulted in a set of 6047 proteins using proteotypic peptides. We went on to compare the two lists and their accompanying fold change values using the gene name as a common identifier for both data types. Standard thresholds of the absolute log2-fold-change above 1 and adjusted *p*-values below 0.05 were applied to identify differentially expressed genes and differentially abundant proteins. A total of 5483 genes/proteins were shared between the two lists. Correlation between relative expression values was calculated with Pearson’s correlation coefficient for the entire dataset, a set of 3608 genes, which were shared between the transcriptome list and the set of peptides with significantly altered abundance levels (at an absolute cut-off value of 0.5), and an even more restricted set of 741 shared genes/peptides significantly at an absolute cut-off value of 1.

### 4.6. SUMO Immunoprecipitations (SUMO-IPs)

For the SUMO-IPs, we used a previously established protocol [43,44]. Briefly, ESC (CGR8) and ESC-PLM cells (#23) were lysed using a denaturing lysis buffer containing 1% SDS. The cell lysate was then diluted to RIPA buffer conditions. Then, 5 mg of total protein lysates from each cell line were incubated with mouse IgG, monoclonal anti-SUMO1, or anti-SUMO2/3 antibody (SUMO1 21C7 and SUMO2 8A2)-coupled beads overnight at 4 °C. SUMO conjugates were eluted with an excess of SUMO epitope, spanning peptides as previously described [43,44,45]. The experiment was performed in two biological replicates. INPUTS (input protein lysate) and eluted proteins from the two experiments were either analyzed by SDS-PAGE followed by Western blotting with the indicated antibodies or sent for proteomic analysis.

### 4.7. SUMO-IP Analysis

LC-MS/MS: Nano-liquid chromatography-mediated separation of the resulting tryptic peptide mixture was carried out using a Ultimate3000 RSLC system. The volume needed for 500 ng of peptides was loaded directly on a pepsep 25-cm-long nano column (1.9 μm 3 beads, 75 µm ID). Peptide separation was achieved in 60 min using 0.1% (vol/vol) formic acid in water (mobile phase A) and 0.1% (vol/vol) formic acid in acetonitrile (ACN) (mobile phase B), starting with a gradient of 7% Buffer B to 35% Buffer B for 40 min and followed by an increase to 45% in 5 min and a second increase to 99% in 0.5 min for 5.5 min, and then kept constant at equilibration at 7% Buffer B for 4.5 min. The gradient flow rate was set to 400 nL/min in the first 10 min of the gradient and 250 nL/min in the main gradient.

The data acquisition was performed in positive mode using a Q Exactive HF-X Orbitrap mass spectrometer (ThermoFisher Scientific, Waltham, MA, USA). MS data were acquired using a data-dependent (DDA) strategy selecting up to the top 12 precursors based on precursor abundance in the survey scan (m/z 350–1500). The resolution of the survey scan was 120,000 (at m/z 200), with a target value of 3 × 10^6^ ions and a maximum injection time of 100 ms. HCD MS/MS spectra were acquired with a target value of 1 × 10^5^ and a resolution of 15,000 (at m/z 200) using an NCE of 28. The maximum injection time for MS/MS was 22 ms. Dynamic exclusion was enabled for 30 s after one MS/MS spectra acquisition. Peptide match was set as preferred. The isolation window for MS/MS fragmentation was set to 1.2 m/z. A lock mass of m/z 445.12003 was used throughout the analysis. Three technical replicas were acquired per biological replicate.

### 4.8. Western Blot (WB)

Cells were lysed in RIPA buffer (25 mM Tris pH 7.6, 150 mM NaCl, 1% NP-40, 1% deoxycholate, 0.1% SDS, and 1 mM PMSF) supplemented with a protease phosphatase inhibitor cocktail (Complete EDTA Free; Roche Applied Science, Indianapolis, IN, USA). Protein concentration was determined using a Bradford assay. Equal amounts of proteins (20 µg) were subjected to SDS/PAGE. Samples were then transferred to a nitrocellulose membrane (Amersham Hybond) and blocked with 5% BSA in TBST, followed by immunoblotting. The SuperSignal West Pico PLUS Chemiluminescent Substrate (Thermo Fisher Scientific, Waltham, MA, USA) was used to detect the signal using a ChemiDoc Imaging System (Biorad, Hercules, CA, USA).

### 4.9. Protein Turnover Measurements

Cycloheximide (CHX) (Sigma-Aldrich, St. Louis, MO, USA) was added to the cultures to a final concentration of 150 μg/mL. At various time points in the CHX treatment, cells were harvested and lysed. Equal amounts of total proteins were subjected to WB analysis.

### 4.10. Luciferase Assays

The TOPflash and Cyclin B1-luc reporters were transfected along with SALL1 1 or CDCA8-expressing plasmids in HEK293 cell lines using calcium phosphate or Lipofectamine™ 2000 (Thermo Fisher Scientific, Waltham, MA, USA). Then, 48 h after transfection, luciferase activities were measured from cell extracts using a Dual-Luciferase Reporter Assay System (Promega, Madison, WI, USA).

### 4.11. Statistical Analysis

All graphs and statistical analyses were performed using GraphPad Prism 8.0.2 software. All graphs represent values as the mean and the standard deviation (mean ± SD) or the standard error of the mean (mean ± SEM), as indicated in the figure legends. A *p*-value < 0.05 was considered a significant difference, determined by two-tailed unpaired *t*-test, one-way ANOVA, or two-way ANOVA comparisons, as indicated in the figure legends. * *p* < 0.05, ** *p* < 0.01, *** *p* < 0.001, **** *p* < 0.0001, n.s (not significant).

## 5. Conclusions

Integrating our proteomics and sumoylomic data, we demonstrated that PML maintains the pluripotency of ES cells through two mechanisms: (1) it increases the protein abundance levels of genes involved in cell cycle progression and self-renewal; and (2) it activates the sumoylation of key regulators involved in pluripotency, chromatin, and cell cycle by increasing the availability and accessibility of sumoylation mediators to these targets.

## Data Availability

Data are available via ProteomeXchange with identifier PXD058024.

## Supplementary Materials

The following supporting information can be downloaded at: https://www.mdpi.com/article/10.3390/ijms26031145/s1.
