# Supplementary material for: Promyelocytic Leukemia Protein (PML) Regulates Stem Cell Pluripotency Through Novel Sumoylation Targets
Source: Int J Mol Sci. 2025 Jan 28;26(3):1145. doi: 10.3390/ijms26031145 (PMC11818296; doi:10.3390/ijms26031145)
Supplement: Supplementary file 1 [file ijms-26-01145-s001.zip › Supplemental Figures_REVISED.pdf]

# Supplementary Materials

## 1. Comparison between the PML-dependent transcriptome and proteome.

**(a)**

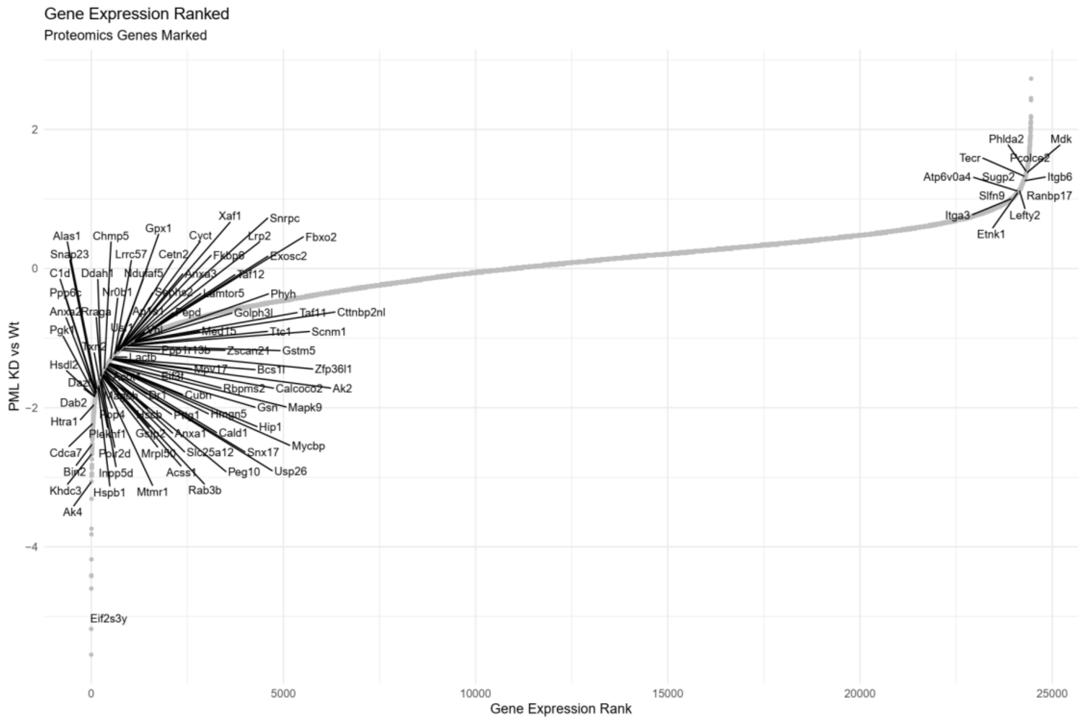


**(b)**

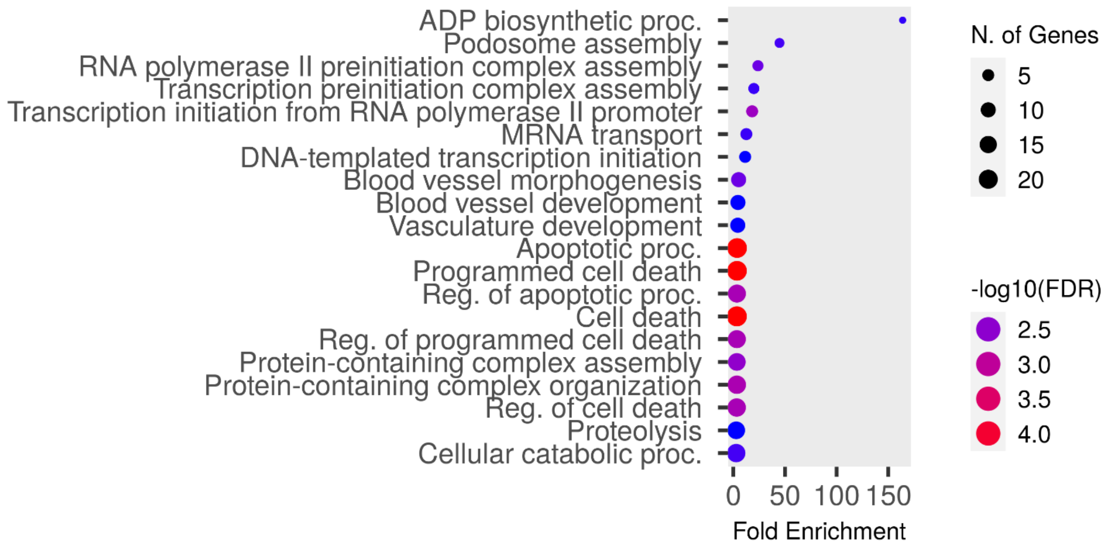


**Figure S1. (a)** Scatterplot of the full ranked list of genes with increasing log2FC value at the transcriptomic level. 96 genes with significant changes at both RNA and protein levels (at a cut-off value of 1) are annotated. **(b)** Functional categories for the list of proteins that were concordantly repressed (Suppl. Table S2.) in PML KD cells in comparison with the WT.

## 2. The majority of PML sumoylation targets are PML-NB associated proteins.

**(a)**

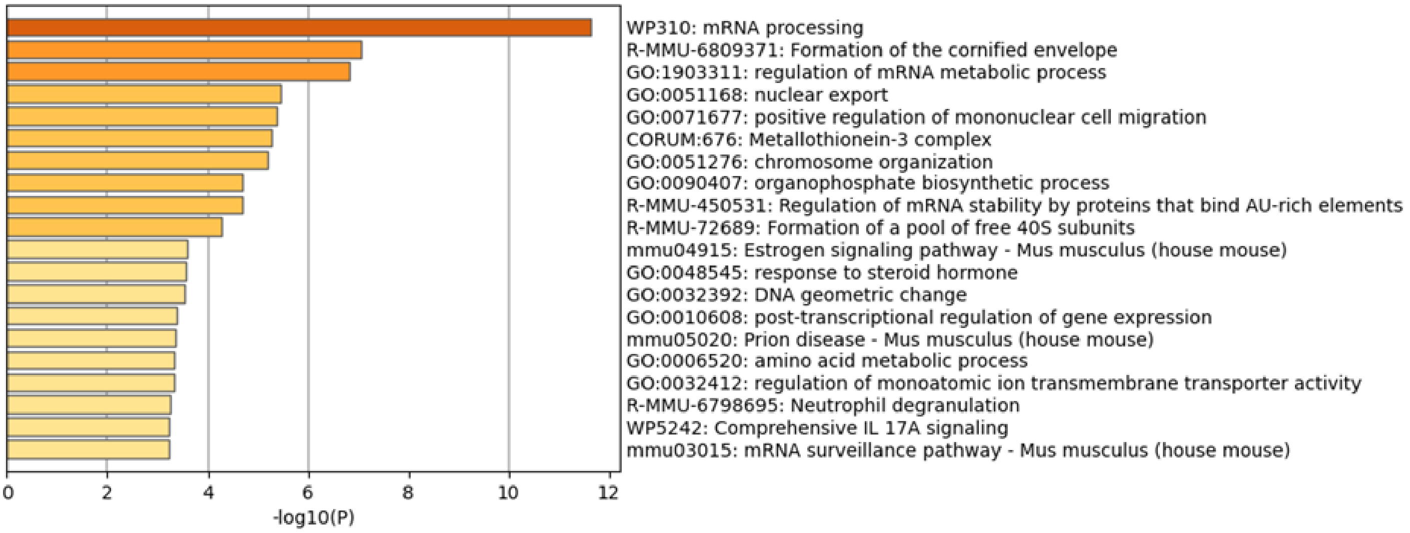


**(b)**

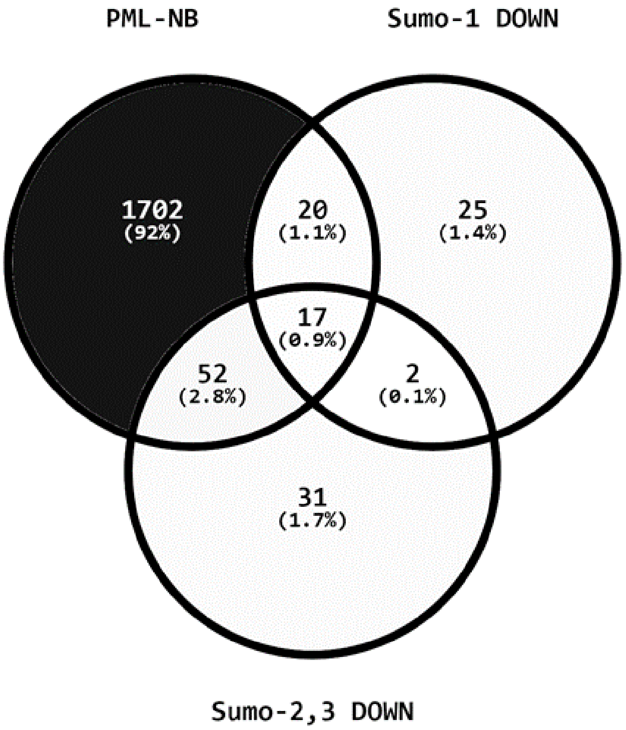


**Figure S2**. **(a)** Enriched terms across genes coding for proteins over-sumoylated in PML KD cells. **(b)** Venn diagram comparing the list of PML-NB client proteins in ES cell (from Sun *et al.*, 2023) [19] with the list of SUMO-1 and SUMO-2,3 proteins that show strong reduction of sumoylation (down-regulated) in PML KD cells.

# 3. PML favors protein sumoylation in ES cells.

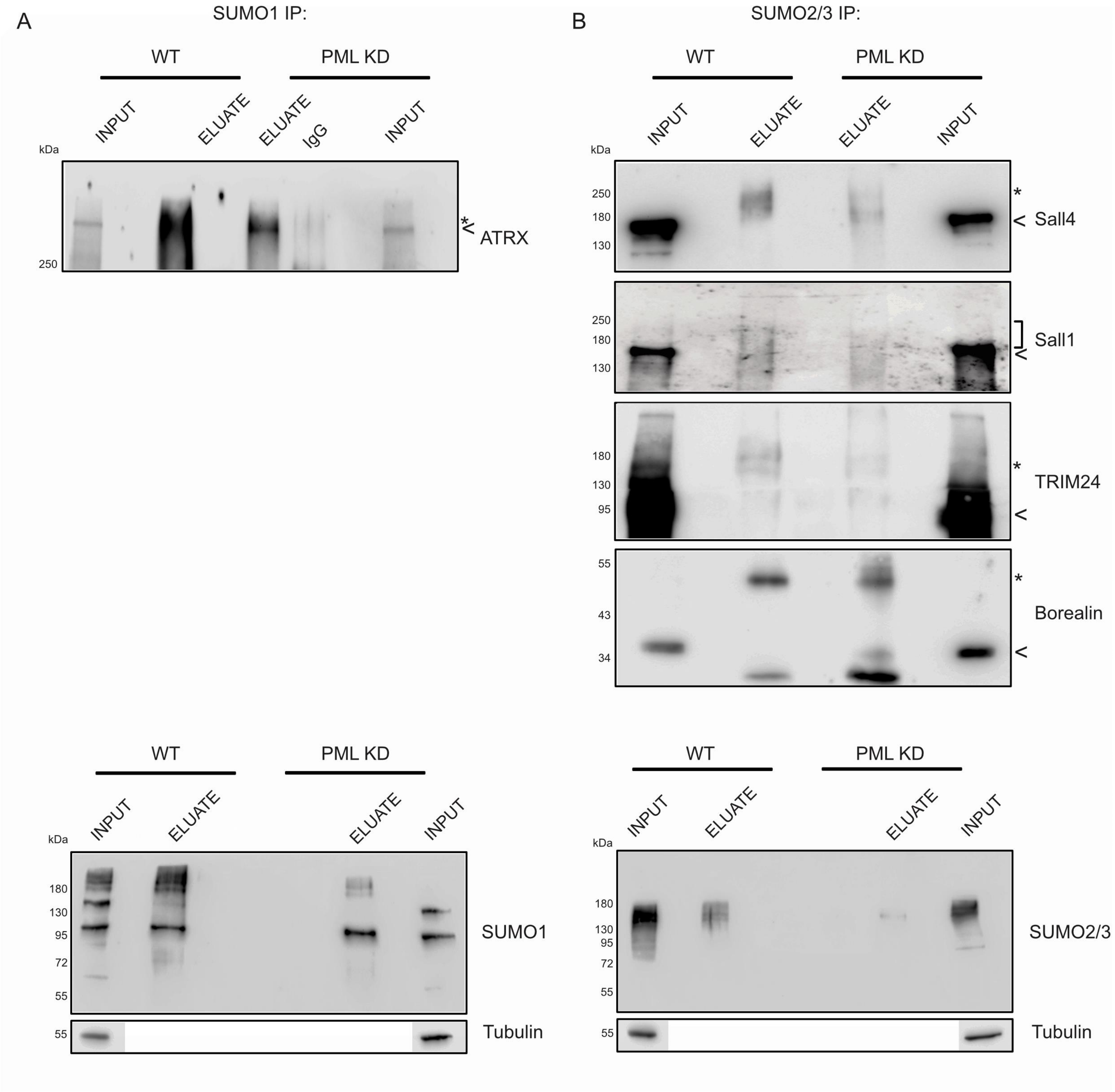


**Figure S3**. Sumoylation validation for selected proteins. Following SUMO-1 or SUMO-2,3 IP of extracts from WT or PML KD cells, SUMO-conjugates were eluted and subjected to WB using the indicated antibodies. **(a)** SUMO-1 IP **(b)** SUMO-2,3 IP

# 4. PML-dependent sumoylation of SALL1 in ES cells

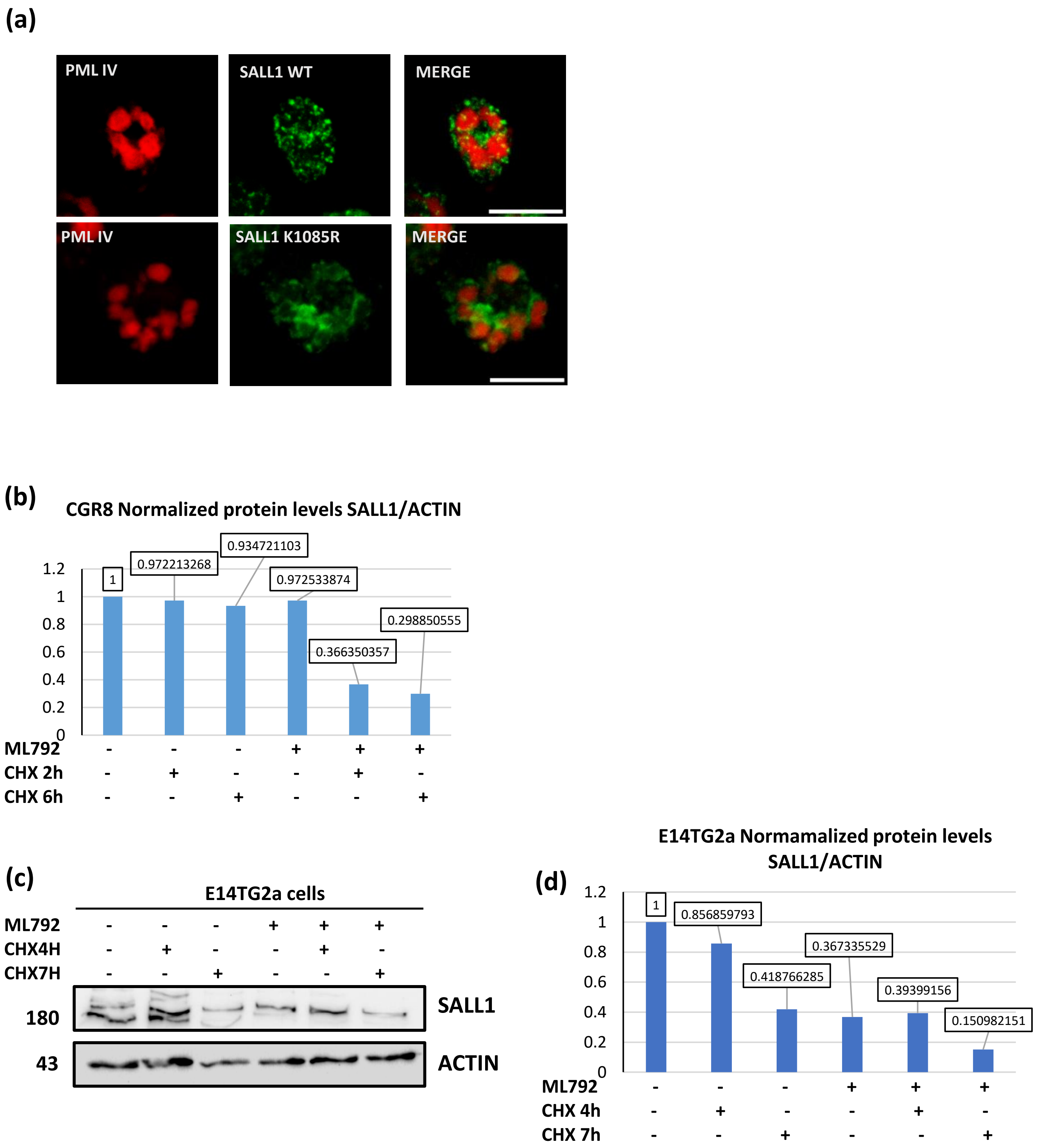


**Figure S4**. (a) Intranuclear localization of SALL1-GFP WT and SALL1$^{K1085R}$–GFP along with PMLIV-mCherry, in HEK293 cells. Scale bar 10μm. (b) Quantification of WB from Figure 4b. (c) The half-life of SALL11 was measured after the addition of 150μg/ml CHX for the indicated points in the absence or presence of ML-792 (1μM) for 24 hrs, in E14TG2a cells. (d) Quantification of WB from Figure S4c.

# 5. PML-dependent sumoylation of CDCA8 in ES cells

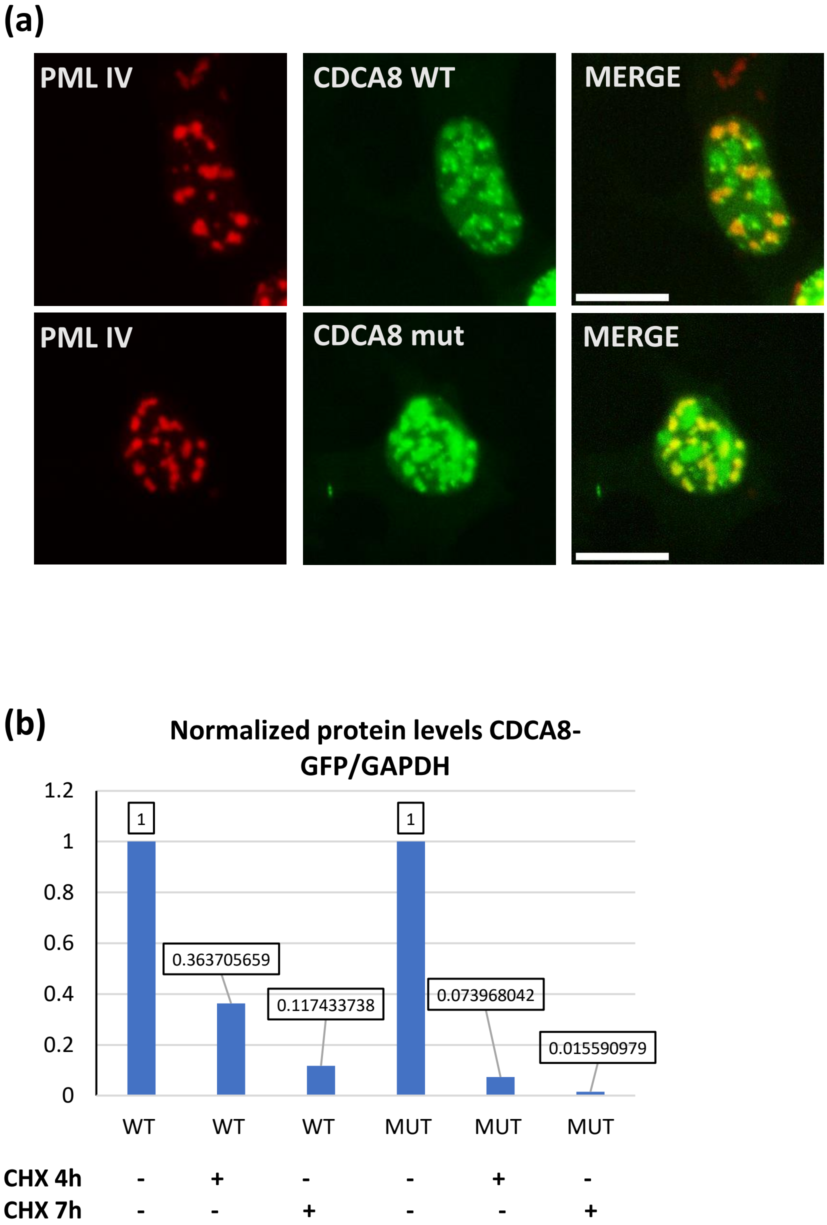


**Figure S5**. (a) Intranuclear localization of CDCA8-GFP WT and CDCA8 mut–GFP along with PMLIV-mCherry, in HEK293 cells. Scale bar 10µm. (b) Quantification of WB from Figure 5a.
